# Future prospects for dissecting inter-individual variability in the absorption, distribution and elimination of plant bioactives of relevance for cardiometabolic endpoints

**DOI:** 10.1007/s00394-019-02095-1

**Published:** 2019-10-23

**Authors:** Rikard Landberg, Claudine Manach, Frederiek-Maarten Kerckhof, Anne-Marie Minihane, Rasha Noureldin M. Saleh, Baukje De Roos, Francisco Tomas-Barberan, Christine Morand, Tom Van de Wiele

**Affiliations:** 1 Department of Biology and Biological Engineering, Food and Nutrition Science, Chalmers University of Technology, 412 96 Gothenburg, Sweden; 2 Université Clermont Auvergne, INRA, UNH, Unité de Nutrition Humaine, CRNH Auvergne, Clermont-Ferrand, France; 3 Center for Microbial Ecology and Technology, Faculty of Bioscience Engineering, Ghent University, Ghent, Belgium; 4 Department of Nutrition and Preventive Medicine, Norwich Medical School, University of East Anglia (UEA), Norwich, UK; 5 University of Aberdeen, the Rowett Institute, Aberdeen, UK; 6 Food and Health Laboratory, Research Group on Quality, Safety, and Bioactivity of Plant Foods, CEBAS-CSIC, Campus de Espinardo, Murcia, Spain

**Keywords:** Plant bioactive compounds, Cardiometabolic, Inter-individual variation, Personalized nutrition

## Abstract

**Purpose:**

The health-promoting potential of food-derived plant bioactive compounds is evident but not always consistent across studies. Large inter-individual variability may originate from differences in digestion, absorption, distribution, metabolism and excretion (ADME). ADME can be modulated by age, sex, dietary habits, microbiome composition, genetic variation, drug exposure and many other factors. Within the recent COST Action POSITIVe, large-scale literature surveys were undertaken to identify the reasons and extent of inter-individual variability in ADME of selected plant bioactive compounds of importance to cardiometabolic health. The aim of the present review is to summarize the findings and suggest a framework for future studies designed to investigate the etiology of inter-individual variability in plant bioactive ADME and bioefficacy.

**Results:**

Few studies have reported individual data on the ADME of bioactive compounds and on determinants such as age, diet, lifestyle, health status and medication, thereby limiting a mechanistic understanding of the main drivers of variation in ADME processes observed across individuals. Metabolomics represent crucial techniques to decipher inter-individual variability and to stratify individuals according to metabotypes reflecting the intrinsic capacity to absorb and metabolize bioactive compounds.

**Conclusion:**

A methodological framework was developed to decipher how the contribution from genetic variants or microbiome variants to ADME of bioactive compounds can be predicted. Future study design should include (1) a larger number of study participants, (2) individual and full profiling of all possible determinants of internal exposure, (3) the presentation of individual ADME data and (4) incorporation of omics platforms, such as genomics, microbiomics and metabolomics in ADME and efficacy studies.

## Key messages

- Human intervention studies are typically too small and do not report data from individuals to allow investigations of relevant determinants of inter-individual variability in ADME and bioefficacy.
- For some plant food bioactive compounds (isoflavones and ellagitannins), particular metabolites are produced only in a subset of the population, i.e., among individuals with a specific metabotype.
- Microbiota is an important determinant of the ADME of many bioactive compounds but microbial gene annotation is often lacking and interference with background diet and temporal variability is high: microbial metabolism capacity is, therefore, difficult to predict.
- Genetic variability is considered an important determinant of the ADME of some bioactive compounds, but there is a large gap in knowledge for many families of plant bioactive compounds regarding biotransformation enzymes and transport proteins.
- Information on other determinants such as age, sex, and diet is too incomplete to make firm conclusions about their impact on the inter-individual variability for most compounds investigated
- Metabotyping individuals appears as essential to increase our understanding and improve prediction of ADME and health effects of plant bioactive compounds. Yet successful stratification examples are scarce and if available (e.g., urolithins), validation studies in larger cohorts are still required.

## Introduction

Cardiometabolic disease, including cardiovascular diseases, type 2 diabetes, obesity, and their risk factors is the leading cause of morbidity and mortality worldwide [1]. It is estimated that patient care and indirect costs represent more than 192 billion euros a year for the EU economy. Population studies have shown that up to 80% of cardiometabolic disease could be prevented through lifestyle changes [2] and that dietary behavior may be most important [3], which offers tremendous public health potential. A large number of observational and interventional studies have provided evidence for the beneficial effects of a diet rich in plant-based foods on cardiometabolic health [4–6]. Although the mechanisms are far from fully understood, bioactive compounds such as polyphenols, carotenoids and phytosterols are being investigated for their health-promoting effects in the context of cardiometabolic diseases. Establishing optimal dose–effect relationships is hampered by the fact that most bioactive compounds have poor to modest bioavailability and there is typically a large inter-individual variation in absorption, distribution, metabolism and excretion (ADME) of such compounds. Moreover, there is also a large inter-individual variability in observed health effects due to inter-individual variation pharmacodynamics parameters, independent from differences in ADME [7]. This clouds the associated health effects from consumption of plant foods and hampers the identification of the effects of particular bioactive compounds in specific subpopulations. Several determinants such as genetic variability, gut microbiota composition, age or sex may explain this inter-individual variability but few studies have collected such information in a way that facilitates a systematic study and conclusions to be drawn. To exemplify, the bioavailability of lutein in healthy human subjects relies on variants in genes encoding proteins involved in carotenoid absorption and metabolism [8]. Another well-known example of a marked inter-individual variation in plant bioactive metabolism is the gut microbial conversion of soy isoflavones into equol and its impact on host physiology [9]. Only 30% of the western population has a microbiota capable of producing equol and these producers are known to gain more health benefits (improved vasomotricity, lower blood LPL and CRP levels) from soy consumption than non-producers [10].

As long as information about determinants of inter-individual variability is not collected and strategies to take them into account are lacking, it is likely that the number of studies with conflicting results will continue to grow. This will prevent policy makers to establish evidence-based dietary guidelines, consumers will not be convinced to adopt the recommended dietary habits and the food and nutraceutical industry will be hesitant to invest in product innovation that gets the highest health benefit from food products and/or targets specific consumer subpopulations.

The main goal of the COST POSITIVe action was, therefore, to build an open European scientific network to tackle the question of inter-individual variation in response to consumption of plant bioactive foods and work with industry and regulatory bodies to translate findings to product innovation and refined dietary recommendations. Within COST POSITIVe, working group 1 (WG1) aimed to improve the understanding of factors affecting inter-individual variation in bioavailability of bioactive compounds from plant-based foods. WG1 brought together scientists from various disciplines: nutritionists expert in the bioavailability of different classes of bioactive compounds, microbiologists, geneticists, epidemiologists, food technologists, and experts in metabolomics [11].

**Fig. 1:**
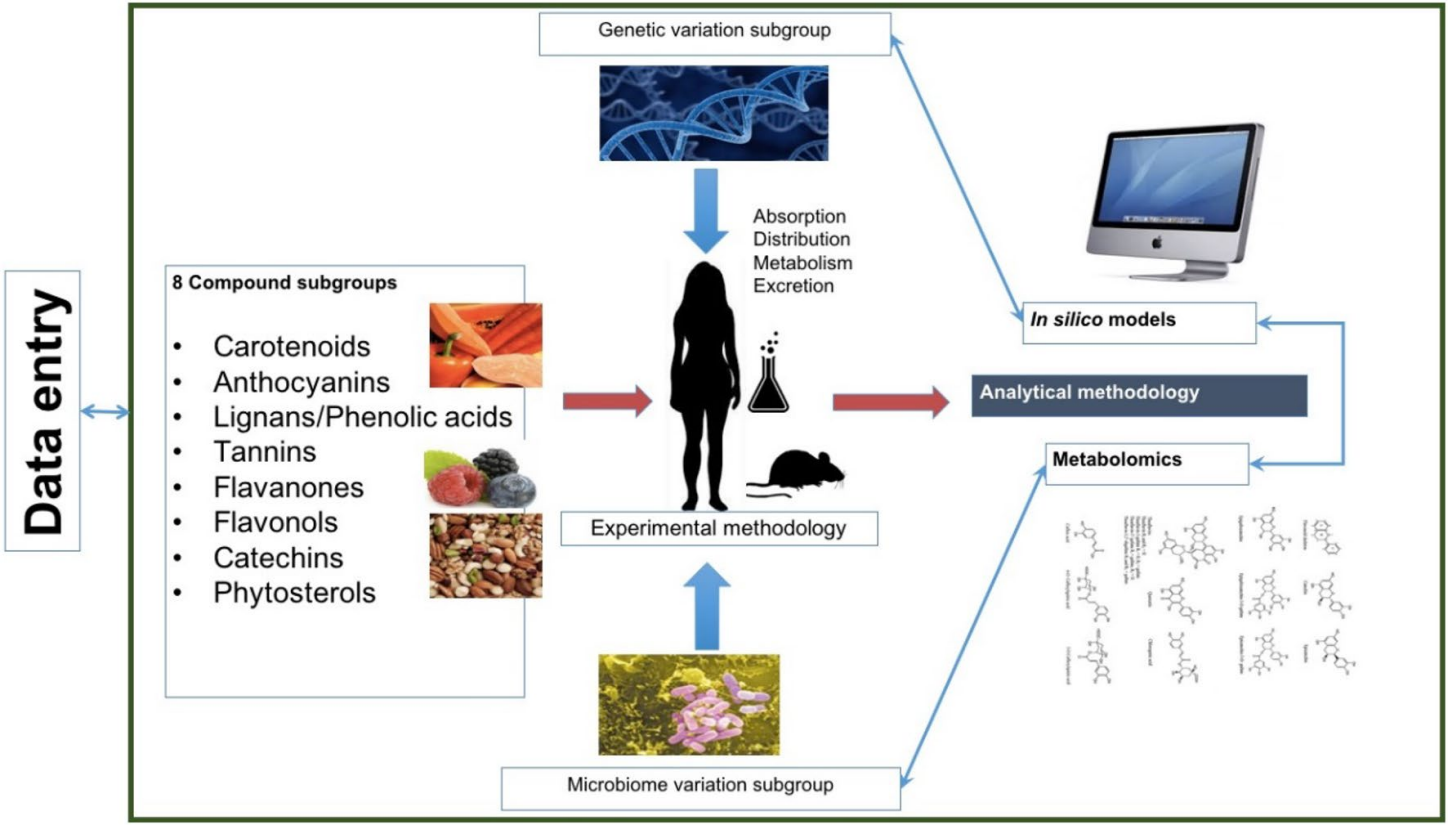
Structure of the COST Positive working group 1 focusing on the inter-individual variability in absorption, distribution, metabolism and excretion of selected plant food bioactive compounds

Processes including absorption, distribution, metabolism and excretion (ADME) of selected bioactive compounds were included when mapping determinants of inter-individual variability. The WG had three aims: (1) to identify the main factors that modulate the ADME of plant food bioactive compounds and to improve methods and tools to assess individual exposure. Bioactive compound classes were selected based on their presence in plant-based foods and to what extent they have been studied and/or have established effects on cardiometabolic risk factors in humans. To address the aim, WG1 established a joint data input structure that was followed for all compound classes and started with a detailed literature data analysis to collect reported determinants of inter-individual variability in ADME after oral intake of selected plant bioactive compounds (Fig. 1). Eight working subgroups were formed to deal with the selected compound classes who processed the literature data according to predefined guidelines and generated one review or opinion paper per compound class. In parallel to the working groups focusing on dissecting factors important for interpersonal variation of ADME of specific compounds, separate working groups were formed to address aim (2) the compilation of existing knowledge to identify key genes and enzymes related to human and gut microbial biotransformation with major importance for interpersonal variability in the ADME of selected bioactive compounds. Finally, aim (3) was to develop a framework for how metabolomics can be applied to analyze and assess true internal exposure to bioactive metabolites at the individual level and interpersonal variability in ADME. These activities were covered by the creation of the metabolomics subgroup.

## Determinants of interpersonal variability

**Fig. 2:**
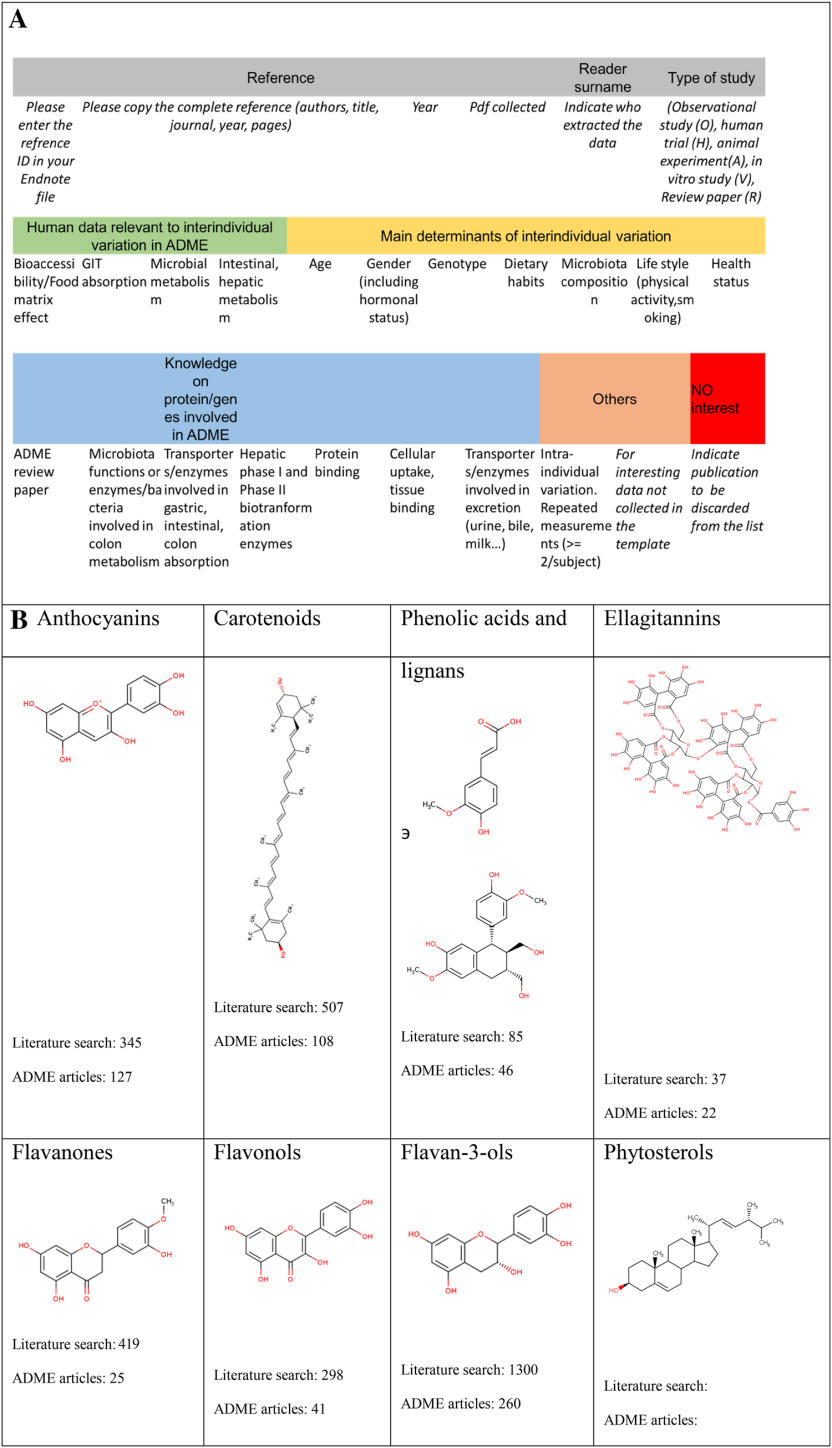
**a** Scheme of possible determinants covered within the literature survey. **b** Molecular structures of the compounds tackled within the literature survey

The first step of WG1 was to select compound classes of interest and to set up the criteria for a literature review to collect the correct information to allow for the dissection of the determinants of inter-individual variability in ADME of selected bioactive compounds. Following a questionnaire in which research interests and expertise were probed, participants organized themselves into different compound subgroups focusing on anthocyanins, carotenoids, lignans and phenolic acids, ellagitannins, flavanones, flavonols, catechins and phytosterols (Fig. 2). A data entry template was established to collate literature information and indicate what articles cover determinants such as age, sex, genetics and microbiome (Fig. 2a). Over 3000 papers were reviewed, from which 511 were eventually selected as the basis to write several reviews. So far, this has resulted in six publications that have been published, accepted or submitted [12–16].

### Lignans

As an example, the literature surveys from lignans resulted in 443 articles fulfilling the search criteria including 96 human studies [15]. The main findings from this extensive literature survey were that variability in plasma levels of enterolactone, one of the main end-products formed by gut microbiota from plant lignans, was large and that gut microbiota and antibiotics were found to be the most important determinants for internal exposure. In addition, age and sex also appeared to be important determinants of plasma enterolactone concentrations and variability. Higher plasma concentrations of enterolactone were found for older individuals and female individuals. This could be primarily explained by the slower gut transit that is typically observed in these aged individuals and the higher lignan intake that was noted for female individuals. Ethnicity also was a determinant of plasma enterolactone and primarily related to altered dietary habits. Finally, a clear link between enterolactone plasma concentrations and health status and lifestyle was observed, with individuals with a high BMI and smoking showing lower circulating plasma levels of enterolactone. The mechanisms behind the effects of specific determinants have not been elucidated in all cases. An important finding from the literature review of lignans was the involvement of gut microbiota in the biotransformation and bioavailability of lignans and its role as a main determinant for plasma enterolactone concentrations. For example, the metabolism of secoisolariciresinol diglucoside (SDG, a major plant lignan) entails subsequent steps of *O*-deglycosylation, demethylation, dehydroxylation and dehydrogenation. Interestingly, not a single microbe is able to completely metabolize SDG into enterodiol and enterolactone: SDG metabolism is always relying on a joint action from different microorganisms [17]. This results in observations that microbiome diversity and composition are clearly correlating with the enterolactone status of human individuals. As the microbiome is largely affected by dietary behavior, it is not surprising that dietary fiber intake associates with a higher microbial diversity and in turn results in higher plasma levels of enterolactone. Conversely, intake of energy and lipids was negatively correlated with plasma enterolactone concentrations.

### Carotenoids

The focus was on lycopene, β-carotene and lutein, which are the three main carotenoid compounds found in blood and tissue. From the factors that are most likely to impact carotenoid uptake, distribution, metabolism and excretion, genetic variations in proteins involved in carotenoid metabolism were the most important [12, 14]. These involve digestion enzymes (PNLIP), metabolizing enzymes (BCO1/2), carrier proteins (SR-B1, CD36, ABCA1 and more) as well as proteins governing secretion into chylomicrons (APOB and MTTP), biotransformation enzymes in the blood and liver and proteins involved in the distribution to target tissues. Interestingly, although gut microbiota are likely not involved in the direct metabolism of carotenoids, they may have an indirect impact on carotenoid ADME through their modulation of bile salt profiles and excretion [12]. As for many other bioactive compounds, determinants such as disease status, body weight and BMI, sex and lifestyle habits (smoking, alcohol consumption) turned out to additionally contribute to inter-individual variability.

### Flavonols

Despite the large impact of inter-individual variability in ADME of flavonols, only 10 out of 55 articles reported the participants’ individual data and none of the studies were actually designed to decipher the underlying determinants of inter-individual variability [13]. Variability was smaller for metabolites resulting from small intestinal digestion and absorption than for metabolites that were derived from colon microbiota. While dietary habits, genetic polymorphisms and microbiota composition are reported to impact variability in flavonol ADME, it is difficult to accurately assess their individual contribution, given the information presented in the publications. As for many bioactive compounds, there is a need for more detailed intervention studies of larger size, collection of more information about the characteristics of the enrolled individuals, such as age, diet, sex, diet, lifestyle and health status, and attention for obtaining individual pharmacokinetic data of quercetin and all of its metabolites.

### Phenolic acids

Phenolic acids are widely present in plant-based foods in free or conjugated forms. Their bioavailability depends on the free/conjugated form and is affected by food processing. Phenolic acids are metabolized both by the host and gut microbiota, resulting in conjugations and structural modifications of the compounds. Only a few studies have investigated inter-individual variability in ADME and health responses to phenolic acid-rich foods and these studies were reviewed and data summarized [18]. As for many other bioactive compounds, phenolic acid metabolite profiles and health responses of phenolic acids show large inter-individual variability, which seems to be related to the metabolic status, sex, dietary habits, and genetic polymorphisms, but the co-ingestion of other dietary bioactive compounds may also contribute. For phenolic acids, it may be of particular interest to design future studies to allow potential confounding effects of other food phytochemicals and their metabolites present in concomitance with phenolic acids to be revealed. Moreover, since phenolic acid metabolites are highly dependent on gut microbiota, a better understanding of the gut-host interplay and microbiome biochemistry is becoming highly relevant in understanding the impact of phenolic acids and their response/non-response in health outcomes.

### Ellagitannins and ellagic acid

These (poly)phenols belong to the family of hydrolysable tannins. They are poorly absorbed in the stomach, small intestine and colon. However, they are extensively metabolized by the gut microbiota to urolithins, which are readily absorbed. Urolithin Phase II metabolites reach plasma concentrations as high as 10 µM [19]. Urolithins show several biological effects on cardiometabolic risk biomarkers [20–22], anti-inflammatory effects at different organs (gut, vascular tissues and neuronal tissues) [23], and some interesting effects recently observed on muscular performance and exercise recovery [20]. Inter-individual variability in the ADME of urolithins has been reported, and three different metabotypes A, B and 0, have been described [19]. These metabotypes associate with the occurrence of specific bacterial strains in the gut and show that gut microbiota composition can be potentially a relevant determinant in the ADME of ellagitannins and ellagic acid, and, therefore, an important determinant of their cardiometabolic health effects. In fact, it has been shown that individuals of metabotype B respond better to the administration of pomegranate ellagitannins leading to significant decreases in cardiometabolic risk biomarkers, while metabotype A does not show the same effects [20]. Specific bacterial strains have been associated with the production metabotype A and B urolithins [24]. However, no matching of metabotypes A, B and 0 with the gut microbiome enterotypes has been observed [25].

### Flavanones

A large inter-individual variability in the ADME of citrus flavanones has been reported and high, medium, and low flavanone metabolites excreters have been identified in different studies after the intake of flavanone rutinosides, citrus flavanone extracts and citrus juices [26–28]. One key factor in this variability is the necessity of gut microbiota to convert the un-absorbable flavanone rutinosides (rhamnosyl(1-6)glucosides, hesperidin, narirutin, naringin, neohesperidin and eriocitrin) to the readily absorbable aglycones (hesperetin, naringenin and eriodictyol). Human intestinal cells do not have the rhamnosidase activity needed, while many gut bacterial species do have rhamnosidase activity, including bifidobacteria, lactobacilli and *Bacteroides* spp. Despite the demonstrated high inter-individual variability in flavanone ADME, no correlation with gut microbiota composition has been studied so far, and no correlation of flavanone absorption with the effects on cardiometabolic health biomarkers has been demonstrated.

## OMICs-strategies for assessment of interpersonal variability

### Metabotypes in the field of plant food bioactives

Individuals that share similar metabolic phenotype, i.e., that may have similar pharmacokinetics and/or bioavailability of plant bioactive compounds and/or similar response patterns of such, may be grouped into*metabotypes*. The general concept of metabotypes was already introduced 20 years ago by Gavaghan et al. [29] proposing a metabonomic approach to relate metabolic balance and metabolite excretion data to host phenotype and genotype. In 2008, the metabotype concept was expanded with a transgenomic approach to link up microbiome profiles with metabolic phenotypes in the host [30] followed by the proposal of Bolca et al. [31] to actively consider gut microbial metabotyping when elucidating health effects from plant bioactives. Yet, the general metabotype concept suffers from its very broad and often subjective definition, given the multitude and variety of study types (epidemiological vs. intervention), metabolic pathways (host vs. microbiome), study objectives and endpoints of interest where it has been considered useful [32]. For plant food bioactives, while most evidence for the existence of separate human metabotypes stems from differences in microbial metabolic potency, human genetic polymorphisms may also significantly contribute to different (metabolic) phenotypes. To exemplify, polymorphisms in the CYP1A2 encoding genes, responsible for caffeine biotransformation, were previously found to lie at the basis for a modulated risk of hypertension and myocardial infarction for those individuals that display the low caffeine-metabolizing phenotype [33, 33, 34]. Riedl et al. [32] made a plea for a stricter metabotype definition, not in the broad sense where it is unrealistic to make a fit-for-all definition, but rather use sub-definitions that are fit-for-purpose. In the context of plant bioactives, we propose to define metabotypes as the manifestation of human population subgroups that have different metabolic phenotypes for phytochemicals, which could be captured by differences in the metabolome profiles in bodily fluids (plasma, urine, feces, tissues, etc.) after the intake of specific plant food or bioactive compounds. Thus, a metabotype can be defined on the basis of a small number of specific metabolites (e.g., equol or urolithin metabotypes) that reflect a particular metabolic capacity and are sufficient to distinguish meaningful subgroups. Besides, metabotyping can be based on wider metabolic profiles, e.g., using untargeted metabolomics, showing the potential to classify subjects in subgroups with distinct internal exposures to bioactive metabolites in fasting state or as a response to a nutritional challenge. Identifying consumers with different metabotypes can be of major importance to identify those that will have particular health benefit from plant food bioactive compounds as well as risk groups of consumption. This could be the starting point for personalized nutrition concepts, and identification of biomarkers of specific metabotypes differentially associated with response/non-response of the intake of bioactive compounds is highly warranted [35]. In addition, basic understanding about the required optimal dose of plant food bioactive compounds that will ensure the best health benefit is currently lacking for most compounds. Phytosterols are an exception as it has been shown that a daily intake of 2 g is needed to lower total- and LDL cholesterol [36]. However, the optimal dose that brings out the best health benefit most likely differs across individuals and metabotypes.

To accelerate the development of personalized nutrition strategies based on metabotyping, simple and high-throughput methods for the analysis of bioactive compounds and their metabolites at large scale are needed, with robust detection, identification and quantification of plant bioactive parent compounds and metabolites. Methods for analytical coverage of representative plant bioactive compounds and metabolites in human biological samples were worked out and tested in different labs involved in the POSITIVe network [37]. Exploration of interpersonal variation according to variations in microbiome composition or gene polymorphisms is two additional fundaments for stratification of individuals. This requires a profound understanding of the biochemical pathways that include biotransformation enzymes and transport proteins, both from the gut microbiome perspective as well as from the human body perspective.

### MS-based metabolomics as a methodology of choice to address plant bioactives

#### Assessing exposure to plant food bioactive compounds

The internal exposure to polyphenols, carotenoids and other plant food bioactive compounds is largely, but not only, dependent on the level of intake of their food sources. As already mentioned above, the internal exposure is also modulated by food matrix effects as well as by intrinsic and environmental factors affecting the ADME capacity of individuals for xenobiotics [38]. For example, the gut microbiome may confer or not to the host the capacity to produce metabolites such as equol or urolithins. Assessing the exposure to plant food bioactive compounds by the measurement of food intakes using dietary questionnaires followed by conversion into intakes of bioactive compounds using food composition tables is thus not reliable enough and may mask potentially existing relationships with health and diseases. Pioneering studies with isoflavones and ellagitannins have shown that in some cases, health benefits associated with plant food consumption may be restricted to population subgroups with particular metabotypes [20, 39]. Profiling of metabolites present in biofluids, rather than estimating the intake through dietary assessment and food composition tables appears as the best approach to reflect the true exposure to bioactive compounds and their metabolites, and is more closely related with pharmacodynamics at any time point. Targeted metabolomics has been used for years to study the bioavailability of bioactive compounds from plants and more recently to assess kinetics concordance with the improvement of health outcomes [40]. Metabolite data are a good basis for stratification of subjects according to their molecular phenotype reflecting the capacity to produce particular metabolites, and, therefore, one defined aspect of the metabotype.

#### Untargeted metabolomics

Untargeted metabolomics offers an even greater potential for a comprehensive phenotyping of internal exposure embracing the diversity of plant food compounds from various families. Furthermore, owing to its exploratory nature, it is a useful approach to bring new knowledge on still unfamiliar metabolites. Although the number of studies using untargeted metabolomics has exploded the last years, it is still a young discipline, which requires improvements and harmonization of its methods and tools. Metabolomics applied in the field of plant food bioactive compounds is facing the same impediments as in other application domains. Analytical options for untargeted metabolic profiling are multiple and not harmonized, and a large proportion of the signals detected cannot be identified. As these are key limitations, the WG1 of this COST Action evaluated and improved the analytical coverage of untargeted methods for plant food bioactives and contributed to the development of databases and tools to facilitate the identification of plant food metabolites in metabolomics profiles.

Plant food bioactives and their derived human metabolites represent a broad variety of chemical structures, with masses from 100 to > 1300 Da and a large spectrum of polarity, from very polar small microbial metabolites to hydrophobic compounds such as carotenoids or phytosterols. Analyzing simultaneously such an array of chemicals, even with an untargeted approach, represents more than a challenge and analytical choices have to be made. Up to now, metabolomics platforms developed their own profiling methods independently, and no standardization effort has been undertaken as yet. Platforms often do not have a precise knowledge of their own coverage until they have analyzed thousands of standards. POSITIVe organized the first international initiative in the field of plant food bioactives, in the form of a multiplatform test comparing the analytical coverage of 11 LC–MS and 2 GC–MS methods currently used by the partners [41]. Results provided insights for optimization and harmonization of methods. A quality control mixture of 12 inexpensive plant food metabolites was proposed to assess the analytical coverage and resolution of any untargeted LC–MS method. Methods of preparation for plasma, urine and other sample types are also key as ranges of metabolites can unintentionally be discarded. Optimization of preparation methods for plant food bioactive compounds is ongoing. Inter-laboratory validated reference methods will be essential to facilitate the comparison of findings across studies.

#### Use of online databases in the identification of plant-based compounds and their metabolites

Untargeted MS profiling methods are widely used but typically, only 5–20% of the detected signals are identified, leaving a vast pool of potential information unresolved. Databases are essential tools in the identification process. They are queried with experimental spectral data and return the chemical structures matching these data, as hypotheses of identification to be further confirmed. Many compound-centered or spectra-centered databases with various contents are available online and must be used in a complementary manner for a wider chemical coverage. Among the most commonly used are HMDB (www.hmdb.ca), Metlin (https://metlin.scripps.edu/), PubChem (https://pubchem.ncbi.nlm.nih.gov/), ChemSpider (www.chemspider.com/), MassBank (http://massbank.eu/), MoNA (http://mona.fiehnlab.ucdavis.edu/), NIST, Golm Metabolome database, LipidMaps (https://www.lipidmaps.org/), mzCloud (https://www.mzcloud.org/), GNPS (https://gnps.ucsd.edu/), ReSpect (http://spectra.psc.riken.jp/) and FooDB (http://foodb.ca/). The detected signals are not easily identified when the corresponding compounds are not yet present in one of these databases, which is the case for many metabolites of less studied food phytochemicals. PhytoHub (http://phytohub.eu/) is an online database conceived to facilitate the identification of food phytochemicals and their metabolites in metabolomics profiles [42]. It contains > 1700 compounds and is continuously updated by invited experts. The literature survey conducted by WG1 of the COST Action POSITIVe led to a major upgrade of PhytoHub. About 200 metabolites of polyphenols not yet recorded in any database were added, associated with the original literature. PhytoHub can now be searched to get the list of metabolites observed or expected in biofluids after consumption of a given food. For example, when searched for apple, a list of 195 metabolites is obtained, along with the information necessary to identify them in urine or plasma metabolomic profiles. In addition, for compounds whose metabolism has not yet been studied in humans, the most likely host and microbial metabolites can be predicted by Biotransformer (http://biotransformer.ca/), a new open-access tool that applies prediction rules elaborated from machine-learning algorithms and expert knowledge including PhytoHub data [43]. Integration of more chemical, biological and spectral data for phytochemical metabolites in metabolomic databases will be key for a better understanding of food phytochemical ADME and associated inter-individual variation.

Obtaining hypotheses of identification for unknowns using databases is one step in the process. Hypotheses must be validated by comparison of experimental MS/MS spectra or retention time with those of authentic standards. Unfortunately, many standards are expensive or not commercially available. A collaborative platform called FoodComEx (http://foodcomex.org/) is facilitating the sharing of standards, and several chemists from POSITIVe already proposed some precious synthesized metabolites of polyphenols there [44]. MS/MS fragmentation is widely used to support or discard hypotheses of identification, while information associated to the retention time has not been considered enough. We evaluated the potential of retention time prediction by PredRet [45]. This online tool can predict the retention time of compounds in a chromatographic system as soon as they have been experimentally determined in other registered chromatographic systems. We compiled retention time data for 471 plant food metabolites in 24 chromatographic systems used in 19 platforms across Europe. Predictions were very accurate, with a median prediction error of 0.03–0.76 min depending on the systems. Such level of precision represents a great help to accelerate the identification process, with the possibility to discard hypotheses outside the predicted retention time window. PredRet is free to use for new contributors, and the number and the accuracy of predictions will increase with the number of shared data.

Coordination of efforts and large collaborative initiatives are essential to improve and harmonize the assessment of individual exposures to plant food bioactive compounds using untargeted metabolomics. The COST Action POSITIVe has been instrumental in building a community with shared interests, which will hopefully continue to interact and expand efforts to collectively address the most important remaining challenges.

### Genetics

As for other phenotypes [46], it is likely that up to 50% of the cardiometabolic response to intake of plant bioactives is attributable to genetic variation. Variation in genes that modulate plant bioactive ADME and bioavailability as well as the pharmacodynamics of the bioactive compounds, are likely to be important. In the general population, about 99.4% of DNA is common between individuals with the remaining 0.6% defining phenotype and response to the environment including dietary exposure. The 2015 output from the *1000 Genome Consortium*, indicated that there are typically 88 million variants in a human genome [47], including gross structural alterations affecting > 1000 bases/nucleotides in the DNA sequence, such as copy number variation, deletions, insertion and inversion, as well as single-nucleotide polymorphisms (SNPs), where a single base in a nucleotide is changed. The identification of which variants are important in modulating human health represents an enormous challenge. As SNPs constitute > 90% of all genetic variability, and are of high relevance for public health, targeted genotyping, where the focus is SNPs in key genes involved in the metabolic pathway of interest, is a widely used approach. In addition, genome-wide association studies (GWAS) are been conducted (https://www.genome.gov/20019523/, http://www.ebi.ac.uk/gwas/), in which genetic information across the genome can be related to a particular trait, e.g., plant bioactive concentrations.

To date the limited investigations for plant food bioactives have taken a candidate gene approach, focusing on one or a small number of variants in a gene encoding for plant bioactive phase I or II metabolism proteins, with the justification for the selected gene variants rarely provided, and the functional consequences of genotype often unknown. Perhaps the most extensively studied genotype relevant to flavonoid ADME, is a Catechol-*O*-methyltransferase (COMT) missense mutation (rs4680), with a G to A base change resulting in a valine to methionine amino acid substitution at position 158 of the protein. This polymorphism is thought to produce a less-stable protein, with a reduced enzyme activity [48]. In human trials, this genotype has been associated with flavan-3-ol/metabolite plasma and urinary concentrations, breast cancer risk and vascular responses following green tea (source of dietary flavan-3-ol) consumption [49–51], although in the *Minnesota Green Tea Trial*, no overall impact of 12-month intervention or no genotype*treatment interactions on adiposity or cardiometabolic health, was observed [52].

Twenty-two isoforms of the *Urine*-*5’*-*diphosphate glucuronosyltransferases (UGTs)* gene superfamily [53] catalyze the transfer of a glucuronic acid from UDP glucuronic acid to a host of endogenous and exogenous compounds including prescribed medications and plant bioactive compounds, affecting their metabolism. The impact of UGT genotypes on the endogenous concentrations of these compounds, the incidence of associated cancers and response to select drugs has been reported (for review see [39]). Although the bi-directional relationship between UGT and plant bioactive has been reported [54, 55], the impact of UGT genotype on plant bioactive concentrations and efficacy remains to be established.

The ongoing COB trial (NCT01922869) is taking a more comprehensive approach and is sequencing 112 genes, involved in all stages of plant bioactive metabolism from digestion to elimination, to establish their impact on flavonoid ADME over 48 h. No human GWAS investigation of plant bioactive metabolism is available.

### Methodological approaches for the identification of gene variants of interest with respect to plant food bioactives

Once genes of interest are identified, large publicly available databases including *HapMap* and more recently the 1000 Genome Project (where the genomes of a large number of people sequenced and their genotypic data stored) are used to select potentially important variants. Their selection should be on the basis of their known or predicted impact on protein concentration, structure or function, or pathogenicity, or variants which tag and act as genetic biomarkers for a particular gene region.

In addition to the gene itself, variants in adjacent regulatory regions, for example transcription factor binding site (TFBS) and enhancers, which can influence gene expression, are also likely to be important. Therefore, variants in genes regions within 10 Kb from the transcription start sites (TSS) and transcription end site (TES) for each gene should be included. The second step is to determine the effect of these variants. The *Variant Effect Predictor* (VEP) [56] tool is used to provide information regarding variants’ locations, consequences (e.g. ,missense or frameshift), minor allele frequencies (MAFs) and pathogenicity prediction established using *Sorting Intolerant from Tolerant* (SIFT), *Polyphen*, and others. The next step is to search publicly available datasets that have records of studies showing associations of the selected variants with disease incidence or biomarkers. Some of the commonly used datasets include GWAS, dbSNP, Humsavar and pharmacogenetic databases.

Using all this gathered information, the final step, is to select a tagging SNP for each haplotype block (250 kb max) together with SNPs in the inter-block regions, to provide a comprehensive list of variants covering the whole gene region. Priorities for SNPs’ selection include:

1. SNPs previously published in online databases,
2. SNPs with predicted pathogenicity established using SIFT and *Polyphen*,
3. SNPs in promotor regions, exons, 3’UTR and regulatory regions, and
4. SNPs with a MAF ≥ 0.5

Such an approach will result in a list of SNPs which can be used to probe human clinical trial and cohort data, for genotype*ADME*phenotype associations.

At present, research is at the early stages of identifying the main genetic determinants of plant bioactive metabolism and efficacy. It is hoped that specific genotypes will emerge which could identify subgroups who are likely to most benefit from increased intake of selected plant bioactive compounds. When evidence is sufficient, such information may be used in routine health examinations and possibly be used for individual dietary recommendations.

### The role of gut microbiota in inter-individual variability of plant bioactives

**Fig. 3:**
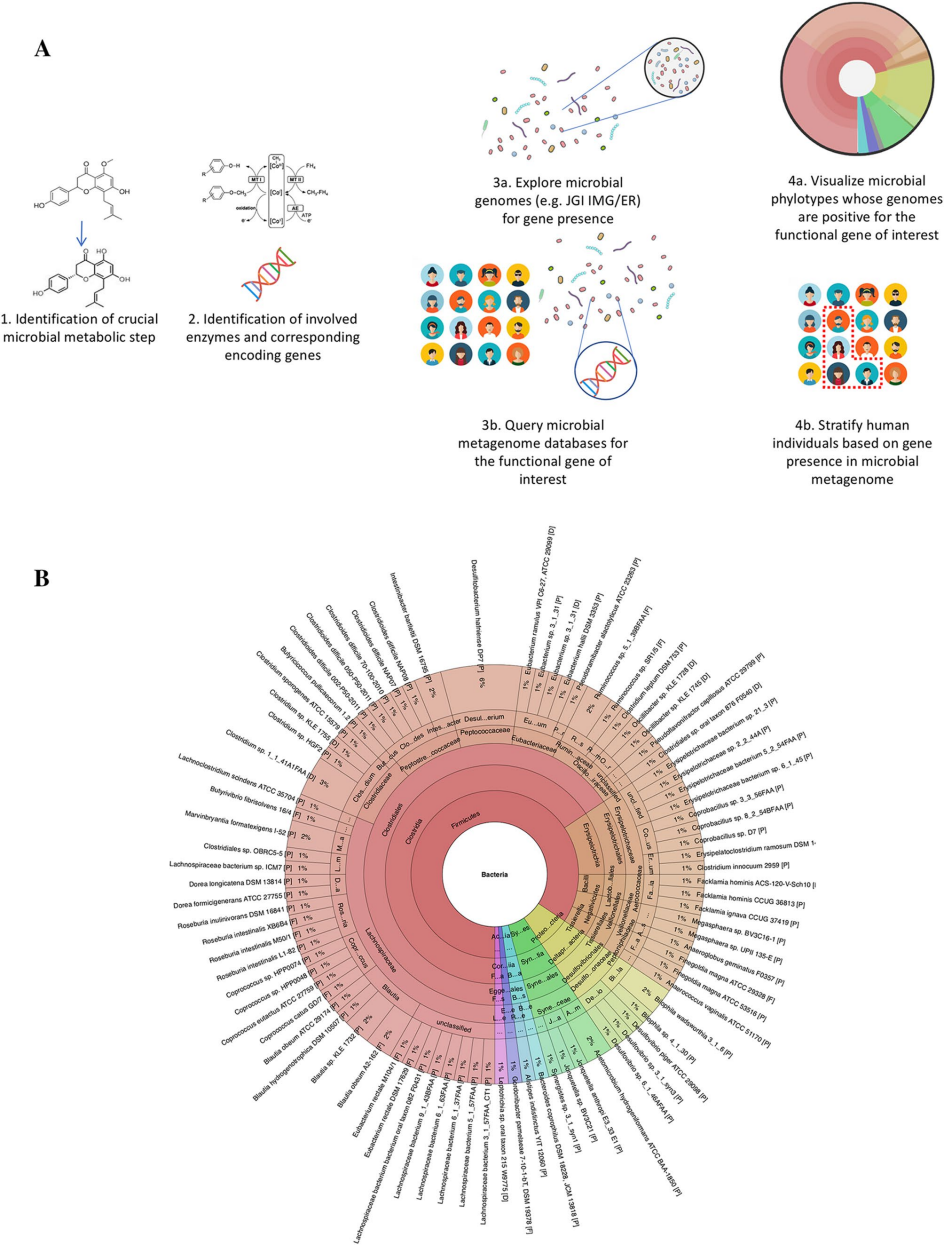
**a** Schematic overview of the workflow to identify genes encoding microbial enzymes responsible for specific metabolic steps of bioactives.**b** Krona™ plot of microbial genomes yielding genes that encode the methyltransferase I component of the *O*-demethylase enzyme system

A thorough understanding of the apparent inter-individual variability in ADME of plant food bioactive compounds is occluded by the lack of insight in the role of gut microbiota in these processes. Gut microbiota can be involved in the breakdown of the food matrix and thus contribute to the bio accessibility of a bioactive which then becomes available for intestinal absorption. In addition, gut microbiota can directly or indirectly (through cleavage of phase-II metabolites) metabolize parent compounds into metabolites with modified bioactivity and susceptibility of intestinal uptake. Finally, alterations in gut microbiome composition and functionality can modulate gut epithelial barrier function resulting in a higher or lower uptake of bioactive molecules of interest. WG1 of the COST Action POSITIVe chose to focus on the microbiome’s metabolic potency and deemed it essential to fully exploit currently available “omics” platforms to study microbial markers of internal exposure and microbial markers of effect. This will enable stratification of human individuals into metabotypes and/or responders based on their microbial metagenome profile (Fig. 3a).

Knowledge of involvement of gut microbiota in the metabolism of plant bioactive compounds requires analytical coverage of possible metabolites and knowledge of metabolic pathways. The groups establishing the reviews of specific bioactive compounds (Fig. 1) were asked to—if at all possible—pinpoint the most crucial step in the (human or microbial) metabolic pathway for the plant bioactive of interest. Identification of microbial genes/gene cassettes of interest that are encoding specialized and unique metabolizing enzymes or transport proteins that may differ amongst individuals depending on their gut microbiome composition was attempted. The search strategy employed was so-called “gene-centric microbial genome and metagenome mining”.

The method consists of (1) screening literature for the crucial microbial metabolic steps that were identified by experts working on the specific compound classes, (2) if absent, screen for microbial enzymes and their encoding genes that are known to perform a comparable metabolic step, (3) use JGI’s (Joint Genome Institute) integrated microbial genome and microbiome (IMG/ER) to filter known HMP (Human Microbiome Project) genomes for these genes (https://img.jgi.doe.gov/imgm_hmp) and export those and (4) use the exported genes to query HMP metagenomes, the 10 million gene catalogue from the former MetaHIT FP7 project or other metagenome databases and subsequently obtain a stratification of human individuals based on their microbiome metagenomic’s information. This approach of stratifying individuals according to their personal microbial (meta)genomic profiles has rarely been attempted before. Several knowledge gaps were identified primarily relate to a lack of accurate gene annotation and database implementation.

Since not all plant bioactive compounds could be covered, a set of model compounds (isoxanthohumol, lignans, flavan-3-ols, ellagitannins, isoflavones, rutin, chlorogenic acid and anthocyanin) was chosen to evaluate employing the gene-centric metagenomic screening strategy. We illustrate our approach with the microbial metabolism of lignans and isoxanthohumol. As mentioned in the section above, enterolactone has been extensively investigated with regard to health effects mainly related to hormonal-dependent cancers. Enterolactone is formed by microbiota-dependent metabolism of plant lignans, in several steps as illustrated by secoisolariciresinol (SECO) diglucoside converted to enterodiol and to enterolactone in the final step. The *O*-demethylation of SECO is a crucial metabolic step in the bioactivation process. *O*-demethylation is not only crucial for lignan activation but also for the conversion of hop-derived isoxanthohumol into its bioactive metabolite 8-prenylnaringenin [57]. Interestingly, *O*-demethylase is a four-component enzyme system that has been well described in several microorganisms [58, 59]. It consists of methyltransferase I, corrinoid protein, methyltransferase II and an activating enzyme. Methyltransferase I typically transfers a methyl group from a substrate (SECO) to the cobalamin (vitamin B_12_)-corrinoid protein while methyltransferase II subsequently transfers the methyl group to tetrahydrofolate, yielding methyl-tetrahydrofolate. The activating enzyme is required to render the corrinoid protein into its appropriate redox status making it in turn receptive again for methylation. While *Eubacterium limosum* ZL-II is known to convert SECO into enterodiol, its genome is unfortunately not completely available. Chen et al. [60], therefore, used the genome of *E. limosum* KIST612 as a reference to further characterize the four-component *O*-demethylase. We used the same reference organism’s proteome and genome to screen the IMG database for available putative *O*-demethylase encoding genes. Interestingly, IMG database exploration for functional protein-encoding genes upstream a metabolic pathway yielded a lower number of positive bacterial genomes. To exemplify, screening for the more general methyltransferase II resulted in positive hits for 2010 bacterial genomes, the corrinoid protein gave 272 bacterial genomes, while methyltransferase I—which probably has higher substrate specificity—only yielded 96 bacterial genomes (Fig. 3b).

This reduction in number of bacterial genomes increases the feasibility of stratifying individuals based on their phylogenetic microbiome composition and making predictions on their probability of harboring a SECO-converting microbiome. A similar gene-centric mining of metagenomic databases can serve as a basis for stratifying individuals based on the presence of functional genes and explore whether specific determinants (ethnicity, diet, health status, sex, age, etc.) form a confounding factor for this stratification. However, this proposal to use gene-centric metagenomic mining does not suffice to identify metabotypes that group individuals based on their ability to generate a high plant-based bioactive concentration in vivo. In-depth and independent validation with separate intervention studies, including the incorporation of all metadata from study participants, need to further prove the usefulness of this approach and indicate to what extent the microbiome is actually involved in a bioactive’s ADME and predict which individuals would experience the largest health effects based on their microbial metagenome.

The identification of putatively involved microbes is still complicated due to some specific issues. The gene-centric metagenomic screening relies on available information of metabolic pathways, which are for many compounds still uncharacterized. Moreover, if functional genes are less well conserved across microbial genomes, gene-centric metagenome mining could yield false negatives depending on the cutoffs that are used. Third, knowledge of gene sequence information does not immediately lead to gene annotation. In such case, KEGG pathways cannot be explored and database screening can only be conducted based on the gene identifier (Gene ID). In addition to the fact that it is difficult to connect an individual’s metagenome with its metadata, it is clear that future research progress in this field will require constantly updated databases and proper gene annotations.

## Conclusions

Available evidence suggests that ADME and bioefficacy of plant food bioactive compounds varies several fold between individuals. Although age, sex, BMI, genotype, the gut microbiome and background diet have been shown to be important for interpersonal variability in selected bioactive compounds, a comprehensive understanding of the individual and interactive contribution of these variables to plant bioactive ADME, and ultimately their impact on tissue function and overall ‘health’, is in its relative infancy. Advancement in current knowledge will require ADME studies, with well-characterized plant food bioactive sources and doses, sensitive metabolomic analysis of tissues and biofluids, along with detailed phenotyping of trial participants for all potentially modulating variables listed above. Such trials should have sufficient sample sizes to draw conclusions regarding variable*ADME associations and derived publications should make data from each trial participant publically available. This would allow integration of data from individual investigations, thereby increasing sample size, and with appropriate statistical approaches facilitating the interactive impact of variables on bioactive ADME to be assessed and modeled. Integrated information may be used to identify metabotypes not only reflecting formation/non-formation of specific metabolites from plant-based bioactive compounds but also of general metabolic phenotype associated with response/non-response to specific bioactive compounds. This could contribute to the development of meaningful dietary recommendations and food product innovation aimed to increase plant food bioactive status and efficacy in all individuals, taking into account the different metabotypes.

## Abbreviations

ADME: Absorption, distribution, metabolism and excretion
ABCA1: ATP-binding cassette transporter 1
APOB: Apolipoprotein B
BC01/2: Beta-carotene oxygenase ½
CD36: Cluster of differentiation 36
COMT: Catechol-*O*-methyltransferase
CRP: C-reactive protein
IMG: Integrated microbial genome
GWAS: Genome-wide association studies
HMP: Human microbiome project
JGI: Joint genome institute
LPL: Lipoprotein lipase
MAF: Minor allele frequency
MTTP: Microsomal triglyceride transfer protein
PNLIP: Pancreatic lipase
WG1: Working group 1
SDG: Secoisolariciresinol diglucoside
SNP: Singe-nucleotide polymorphism
SR-B1: Scavenger receptor class B type 1
UGT: Urine-5’-diphosphate glucuronosyltransferases
TFBS: Transcription factor binding site
TSS: Transcription start sites
TES: Transcription end site
VEP: Variant effect predictor
SIFT: Sorting intolerant from tolerant
